# Effects of Semaglutide on Cardiometabolic Risk in People with Obesity, With and Without Type 2 Diabetes: A Retrospective Observational Study

**DOI:** 10.3390/jcm15124421

**Published:** 2026-06-07

**Authors:** Loredana Deaconu, Oana Albai, Bogdan Timar, Adina Braha, Simona Popescu, Teodora Sorescu, Laura Gaita, Liana Iordan, Romulus Timar

**Affiliations:** 1 Doctoral School of Medicine, “Victor Babes” University of Medicine and Pharmacy, 300041 Timisoara, Romania; loredana.deaconu@umft.ro (L.D.); liana.iordan@umft.ro (L.I.); 2 Second Department of Internal Medicine,” Victor Babeș” University of Medicine and Pharmacy, 300041 Timișoara, Romania; bogdan.timar@umft.ro (B.T.); braha.adina@umft.ro (A.B.); popescu.simona@umft.ro (S.P.); sorescu.teodora@umft.ro (T.S.); gaita.laura@umft.ro (L.G.); timar.romulus@umft.ro (R.T.); 3 Department of Diabetes, “Pius Brînzeu” Emergency County Hospital, 300723 Timisoara, Romania; 4 Center for Molecular Research in Nephrology and Vascular Disease, “Victor Babes” University of Medicine and Pharmacy, 300041 Timisoara, Romania

**Keywords:** obesity, semaglutide, type 2 diabetes, insulin resistance, visceral adiposity, cardiometabolic risk

## Abstract

**Background and Objectives**: Obesity and insulin resistance are major contributors to cardiometabolic disease and type 2 diabetes mellitus (T2DM). This study evaluated the real-world effects of semaglutide on metabolic parameters, body composition, and cardiometabolic risk factors in people with obesity, with and without T2DM, and explored predictors of treatment response. **Materials and Methods**: This retrospective longitudinal observational study included 70 adults with obesity (42 with T2DM and 28 without T2DM) treated with semaglutide according to current clinical guidelines. The primary outcomes were changes in body weight, waist circumference, fasting plasma glucose, and glycated hemoglobin (HbA1c). Secondary outcomes included changes in lipid profile, insulin resistance indices, inflammatory markers, hepatic parameters, and body composition assessed by bioelectrical impedance analysis (InBody770). **Results**: Semaglutide treatment was associated with significant reductions in body weight (−9 kg), waist circumference (−8 cm), HbA1c (−1.1%), systolic blood pressure (−7.5 mmHg), visceral fat area (−30.1 cm^2^), and insulin resistance markers. Improvements in glycemic parameters were more pronounced in participants with T2DM. Skeletal muscle mass (SMM) was relatively preserved during treatment. Baseline HbA1c and visceral adiposity were independently associated with metabolic response. **Conclusions**: In this real-world observational cohort, semaglutide was associated with significant improvements in metabolic parameters, body composition, and cardiometabolic risk markers in people with obesity, with and without T2DM. Baseline metabolic characteristics may influence treatment response.

## 1. Introduction

Obesity and insulin resistance are central drivers of the cardiovascular-kidney-metabolic (CKM) continuum, linking cardiovascular disease, chronic kidney disease (CKD), metabolic dysfunction-associated steatotic liver disease (MASLD), and type 2 diabetes mellitus (T2DM). Visceral adiposity and metabolic dysfunction promote chronic inflammation, endothelial dysfunction, and progressive cardiometabolic damage, highlighting the need for integrated therapeutic strategies targeting both weight reduction and metabolic risk [1,2].

In this context, weight control is a key element of preventive care: weight loss reduces visceral fat, enhances insulin sensitivity, decreases inflammation, improves hepatic steatosis, and slows the progression of CKD and reduces cardiovascular risk [3,4,5]. 

Semaglutide, a long-acting glucagon-like peptide-1 receptor agonist (GLP-1RA), has become a key therapeutic agent that targets multiple components of this interconnected network through pleiotropic and disease-modifying effects. Semaglutide binds to the GLP-1 receptor, a G protein-coupled receptor that activates adenylate cyclase, increasing intracellular cyclic adenosine monophosphate (cAMP) and triggering downstream signaling via protein kinase A (PKA) and exchange protein activated by cAMP [6]. 

At the pancreatic level, semaglutide enhances glucose-dependent insulin secretion while suppressing inappropriate glucagon release from α-cells. This dual hormonal regulation improves postprandial glucose control and reduces fasting hepatic glucose production without increasing the risk of hypoglycemia. Chronic hyperglucagonemia and compensatory hyperinsulinemia are major contributors to insulin resistance (IR) and atherogenic dysmetabolism. By restoring a more physiological insulin–glucagon balance, semaglutide reduces β-cell stress, limits lipotoxicity and glucotoxicity, and preserves endogenous insulin secretion capacity, thereby slowing progression from IR to overt T2DM [7]. 

Central nervous system effects are essential to semaglutide’s ability to reduce weight and improve insulin sensitivity. GLP-1 receptors in the hypothalamus and brainstem regulate appetite, satiety, and energy intake by activating proopiomelanocortin/cocaine- and amphetamine-regulated transcript (POMC/CART) neurons and suppressing neuropeptide Y/agouti-related peptide (NPY/AgRP) pathways. Semaglutide also modulates mesolimbic dopaminergic reward circuits, reducing hedonic eating behavior [8]. 

Visceral adiposity is a key driver of IR because it releases more free fatty acids, proinflammatory cytokines, and harmful adipokines. Semaglutide-induced weight loss improves adipose tissue function by reducing macrophage infiltration, shifting macrophage polarization from a proinflammatory M1 profile to an anti-inflammatory M2 profile, and decreasing secretion of tumor necrosis factor-α, interleukin-6, and resistin. At the same time, semaglutide increases circulating adiponectin, which enhances insulin sensitivity by activating AMP-activated protein kinase (AMPK) and promoting fatty acid oxidation in the liver and skeletal muscle [9,10].

Hepatic insulin resistance plays an important role in linking obesity with dysglycemia, metabolic dysfunction-associated steatotic liver disease (MASLD), and atherogenic dyslipidemia. Although hepatocytes express few GLP-1 receptors, semaglutide indirectly improves hepatic metabolism by reducing glucagon signaling, decreasing free fatty acid flux, and enhancing peripheral insulin sensitivity. These effects contribute to reduced hepatic fat accumulation and improvements in inflammatory and fibrotic markers observed in clinical studies [11]. 

IR is closely linked to endothelial dysfunction, a key early step in atherosclerosis and CVD. GLP-1 receptor activation in endothelial cells increases nitric oxide synthase activity, boosts nitric oxide bioavailability, and reduces oxidative stress by inhibiting reactive oxygen species (ROS) production. These effects enhance insulin-mediated vasodilation, reduce arterial stiffness, and lessen vascular inflammation. Semaglutide also lowers blood pressure and improves lipid profiles by reducing triglycerides, small dense LDL particles, and postprandial lipemia, thereby further reducing atherogenic risk [12]. 

Within the CKM continuum, renal involvement increases cardiovascular risk. Obesity and IR promote glomerular hyperfiltration, sodium retention, and inflammatory and fibrotic pathways, thereby accelerating CKD progression. Semaglutide improves renal function through multiple mechanisms, including weight loss, reduced blood pressure, improved glycemic control, and direct anti-inflammatory effects on the renal microvasculature. Clinical data show reductions in albuminuria and a slower decline in estimated glomerular filtration rate, supporting a nephroprotective role that, in turn, indirectly reduces cardiovascular morbidity and mortality [13]. 

Large cardiovascular outcomes trials have confirmed that the molecular and physiological effects of semaglutide translate into meaningful reductions in risk. The SUSTAIN-6 (Trial to Evaluate Cardiovascular and Other Long-term Outcomes with Semaglutide in Subjects with Type 2 Diabetes) and SELECT (Semaglutide Effects on Cardiovascular Outcomes in People with Overweight or Obesity) trials showed significant decreases in major adverse cardiovascular events, including myocardial infarction and stroke, even in populations without diabetes, highlighting the key role of IR, obesity, and inflammation as modifiable indicators of CVD [14,15]. 

The aim of this study was to investigate, under real-life conditions, the impact of semaglutide treatment on anthropometric, glycemic and cardiometabolic parameters in obese patients, regardless of the presence of T2DM, as well as to identify changes in body composition and determinants of metabolic response to therapy.

## 2. Materials and Methods

### 2.1. The Study Design and Patients

The retrospective longitudinal observational study included 70 adult patients who were assessed and treated in a specialized metabolic and obesity management program at “Pius Brînzeu” Emergency Hospital, Timisoara, from July 2025 to February 2026. Patients were eligible for inclusion in the study if they met the following criteria: age ≥ 18 years, body mass index (BMI) ≥ 30 kg/m^2^, and T2DM status, allowing for a comparative observational analysis. Exclusion criteria included individuals under 18 years of age, pregnant or breastfeeding women, individuals with severe liver or kidney failure, active cancer, severe anemia, uncontrolled endocrine disorders other than diabetes, or conditions that could affect metabolic or body composition assessments. Patients with secondary causes of obesity or severe acute illnesses were also excluded. This study was conducted and reported in accordance with the STROBE (Strengthening the Reporting of Observational Studies in Epidemiology) statement/guidelines.

Among the participants, 42 patients had a confirmed diagnosis of T2DM, and 28 were non-diabetic at baseline. Post hoc power analysis indicated that with 70 patients (42 T2DM, 28 non-T2DM), the study had >80% power to detect a clinically meaningful difference in weight change of 5 kg between groups (α = 0.05, two-tailed), based on observed standard deviations. Patients were stratified by diabetes status (diabetic vs. non-diabetic) and sex for comparative analysis.

All participants received injectable semaglutide administered once weekly according to current clinical recommendations and routine therapeutic protocols for obesity and T2DM management. Treatment was initiated at 0.5 mg/week, followed by progressive dose escalation to 1 mg/week and subsequently to 1.7 mg/week, depending on treatment tolerability and clinical response.

**Figure 1:**
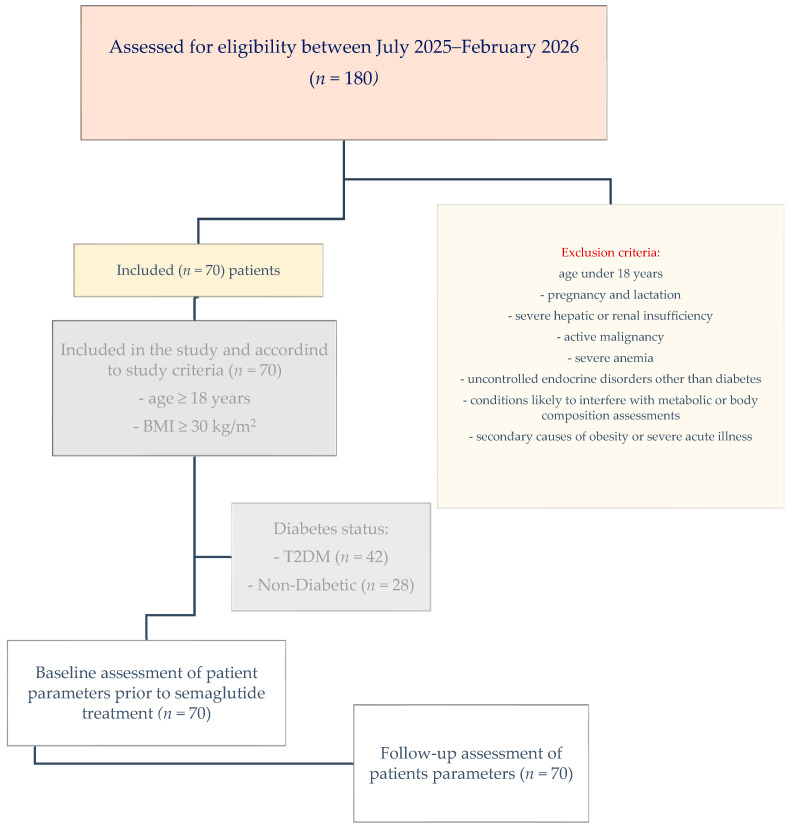
STROBE diagram of the study design.

The average age of the study population was approximately 52 years, with both female and male participants. All patients were classified as having obesity based on BMI and evaluated for metabolic health, body measurements, and body composition at baseline (T0) and follow-up (T1). Patients with T2DM had a confirmed diagnosis before study entry, whereas non-diabetic participants did not meet the diagnostic criteria for diabetes at baseline. All participants were assessed according to standardized clinical protocols, including regular metabolic monitoring and lifestyle counseling. The STROBE (Strengthening the Reporting of Observational Studies in Epidemiology) diagram is shown in Figure 1. 

The study protocol was approved by the Local Ethics Committee for Scientific Research at “Pius Brînzeu” Emergency Hospital, Timisoara (approval No. 518/30 December 2024). The investigations were conducted in accordance with the Declaration of Helsinki. Written informed consent was obtained from all participants before inclusion in the study.

### 2.2. Study Objective

The primary objective of this study was to evaluate the effects of semaglutide on body weight, glycemic control, and cardiometabolic risk parameters in people with obesity, with and without T2DM.

Secondary objectives included assessing changes in lipid profile, insulin resistance, inflammatory markers, hepatic steatosis and fibrosis indices, and body composition parameters, particularly visceral adiposity and SMM preservation. The study also explored potential predictors of metabolic and glycemic response to semaglutide treatment.

### 2.3. Medical Assessments

To achieve the study objectives, the real-world benefits of semaglutide treatment in reducing cardiovascular risk and preventing T2DM were assessed by analyzing changes in anthropometric, metabolic, and body composition parameters in patients with obesity, with and without T2DM. “Real-world” refers to patients managed in routine clinical practice within a specialized obesity and metabolic disease program, without protocol-driven interventions beyond standard medical care.

Demographic data included age, sex, and diabetes status. Anthropometric assessments included body weight, BMI, and waist circumference (WC), measured at baseline and at follow-up visits.

Body composition was assessed using multifrequency and segmental bioelectrical impedance analysis with the InBody770 analyzer (InBody Co., Ltd., Seoul, Republic of Korea), a noninvasive device that measures tissue resistance and reactance in response to low-intensity electrical currents. This method allowed estimation of SMM, total fat mass, body fat percentage, visceral fat area (VFA), total body water, and basal metabolic rate. Segmental analysis provided information on the distribution of muscle and fat in the limbs and trunk, enabling assessment of treatment-related changes in visceral fat and muscle mass preservation.

The effect of treatment on glycemic control was evaluated by analyzing changes in fasting plasma glucose and glycated hemoglobin (HbA1c). Metabolic responses were compared between patients with and without diabetes to assess the potential of semaglutide to prevent progression to T2DM.

Lipid profile assessment included triglycerides, high-density lipoprotein cholesterol (HDLc), and low-density lipoprotein cholesterol (LDLc). Changes in these parameters served as indirect markers of cardiovascular risk. Additionally, derived metabolic indices, such as the triglyceride-to-HDL cholesterol ratio (TG/HDLc) and the triglyceride–glucose (TyG) index, were calculated.

Systemic inflammatory status was assessed by measuring C-reactive protein (CRP), and changes were analyzed in relation to HbA1c. Patients included in the study were clinically stable during the evaluation period, without evidence of acute infection or systemic inflammatory conditions that could significantly influence inflammatory markers. No patients receiving chronic systemic corticosteroid therapy were identified during follow-up.

Renal function was assessed by measuring serum creatinine, estimating the glomerular filtration rate, and calculating the urinary albumin-to-creatinine ratio.

Metabolic liver involvement was assessed using liver biochemical markers, including alanine aminotransferase (ALT), aspartate aminotransferase (AST), gamma-glutamyl transferase (GGT), and serum albumin (ALB). Non-invasive indices of hepatic steatosis and fibrosis were calculated from available parameters, and changes in these indices were used to evaluate the effect of semaglutide treatment on liver fat accumulation and fibrosis.

Insulin resistance and cardiometabolic risk were further evaluated using the TG/HDLc ratio, the TyG index, HOMA-IR, and the metabolic score for insulin resistance (METS-IR). To identify potential predictors of glycemic and cardiovascular responses to treatment, associations between baseline anthropometric, body composition, and metabolic parameters and treatment-induced changes were assessed. The definitions of hepatic, metabolic and insulin resistance indices are presented in Table 1.

### 2.4. Statistical Analysis

Statistical analyses of the characteristic groups were conducted using MedCalc Software Ltd., Ostend, Belgium; https://www.medcalc.org; 2025 (accessed on 17 April 2026). A *p*-value threshold of 0.05 was used for statistical significance, corresponding to a 95% confidence level. Continuous variables were assessed for normality using the Shapiro–Wilk test. Non-normally distributed variables are reported as median (interquartile range, IQR). Categorical variables are expressed as absolute numbers and percentages.

Comparisons between two independent groups (women vs. men; patients with diabetes vs. those without diabetes) were performed using Student’s *t*-test for normally distributed variables and the Mann–Whitney U test for non-normally distributed variables. Categorical variables were compared using the chi-square (χ^2^) test.

To evaluate treatment-related changes from baseline to follow-up (pre-post analysis), the Wilcoxon signed-rank test was used. Changes from baseline to follow-up were calculated as Δ = follow-up-baseline. Correlation analyses among metabolic, glycemic, anthropometric, and body composition parameters were performed using Pearson correlation for normally distributed variables and Spearman rank correlation for non-normally distributed variables.

## 3. Results

### 3.1. Study Population and Baseline Characteristics

A total of 70 patients were included in the study; 28 (40.0%) had obesity without T2DM (primary prevention group), and 42 (60.0%) had obesity with established T2DM (secondary prevention group). Table 2 summarizes baseline demographic, anthropometric, metabolic, cardiovascular, hepatic, and body-composition characteristics. Patients with T2DM were significantly older (*p* < 0.05) and had a higher baseline cardiometabolic risk profile, including higher body mass index, waist circumference, blood pressure, fasting plasma glucose, HbA1c, atherogenic lipid levels, inflammatory markers, and indices of hepatic steatosis and fibrosis. Baseline SMM and total fat mass did not differ significantly between groups.

### 3.2. Semaglutide Doses, Duration, and Relation with Body Weight

The median duration of semaglutide treatment was 6.5 months [5.5–7.5] in the NoT2DM group and 8.5 months [6.0–9.0] in the T2DM group (*p* < 0.001, Mann–Whitney test). Both groups received titration doses of 0.5 mg (4.3%), 1 mg (18.6%), and 1.7 mg (77.1%). A chi-square test of independence showed no significant association (χ^2^ = 3.09, *p* = 0.21) (Table 3). Overall, 97.1% of patients achieved at least 5% weight loss, and 42.9% achieved at least 10% weight loss. Of these, 95.2% of patients with T2DM and all patients without T2DM achieved at least 5% weight loss (chi-square test, χ^2^ = 1.35, *p* = 0.24), and 50% of patients with T2DM and 32.1% of patients without T2DM achieved at least 10% weight loss (chi-square test, χ^2^ = 2.15, *p* = 0.14). Regarding sex, 95.5% of males and 97.9% of females achieved at least 5% weight loss (chi-square test, χ^2^ = 0.35, *p* = 0.56), and 36.4% of males and 45.8% of females achieved at least 10% weight loss (Table 4). 

A multiple linear regression was conducted to assess the association of treatment duration and administered dose with weight change. The results were statistically significant (F = 20.88, *p* < 0.0001) and explained 38.4% of the variance in weight change (R^2^ = 0.384; adjusted R^2^ = 0.366). Both predictors were significantly associated with weight change. Treatment duration had a positive effect (β = 0.1618, *p* = 0.0056), indicating that longer treatment duration was associated with greater weight change. The administered dose showed a stronger positive association (β = 4.2599, *p* < 0.0001), making it the main predictor in the model. Residuals were normally distributed (Shapiro–Wilk test, *p* = 0.8689), confirming that model assumptions were met.

### 3.3. Changes from Baseline (T0) to Follow-Up (T1)

**Figure 2:**
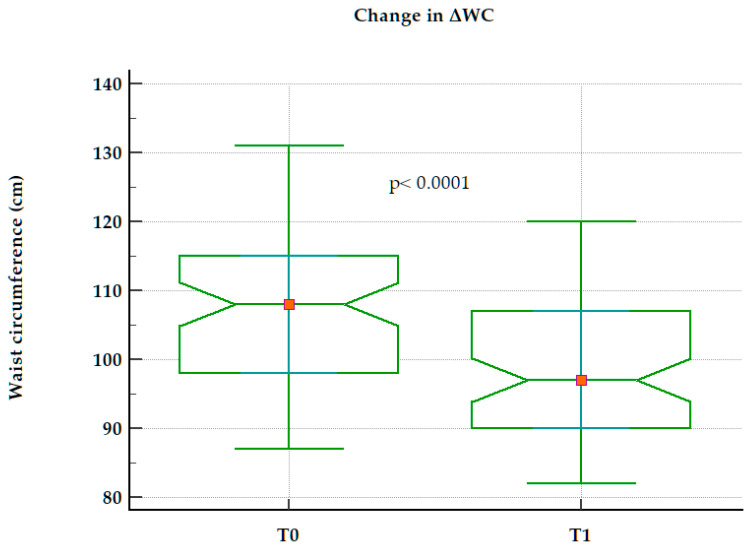
Treatment efficiency: comparison of waist circumference reduction between the two assessment points (T0, baseline; T1, follow-up). WC is presented as the median (square markers) with 95% confidence intervals for the median (vertical bars).

**Figure 3:**
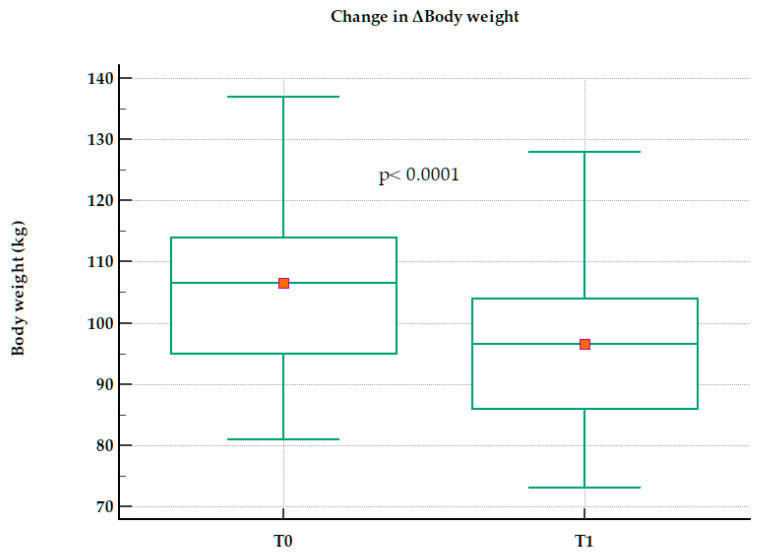
Treatment efficiency: comparison of body weight reduction between the two assessment points (T0, baseline; T1, follow-up). Body weight is presented as the median (square markers) with 95% confidence intervals for the median (vertical bars).

Across the cohort, semaglutide treatment was associated with significant improvements in body weight, body mass index, waist circumference, fasting plasma glucose, HbA1c, insulin resistance (HOMA-IR), blood pressure, atherogenic lipid parameters, and C-reactive protein (Table 5). Between T0 and T1, most evaluated parameters showed statistically significant improvements (Wilcoxon signed-rank test, *p* < 0.0001), except for ALB (*p* = 0.16). Improvements in waist circumference and body weight, with Hodges–Lehmann median differences of −8.0 cm (95% CI: −9.0 to −7.5) and −9.5 kg (95% CI: −10.0 to −8.5), respectively, are shown in Figure 2 and Figure 3.

**Figure 4:**
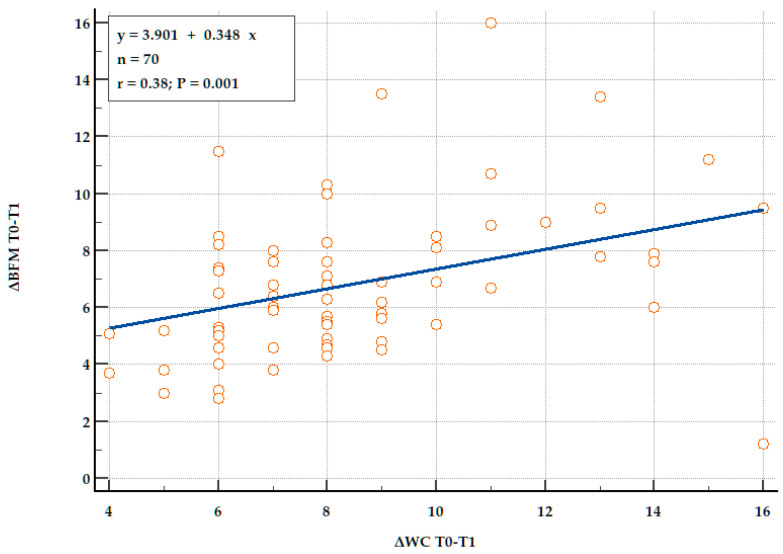
Scatter plot showing the relationship between changes in waist circumference (ΔWC) and body fat mass (ΔBFM). The blue solid line represents the linear regression fit (r = 0.36, *p* = 0.001; R^2^ = 0.142; n = 70) and the red circles represent an individual participant.

**Figure 5:**
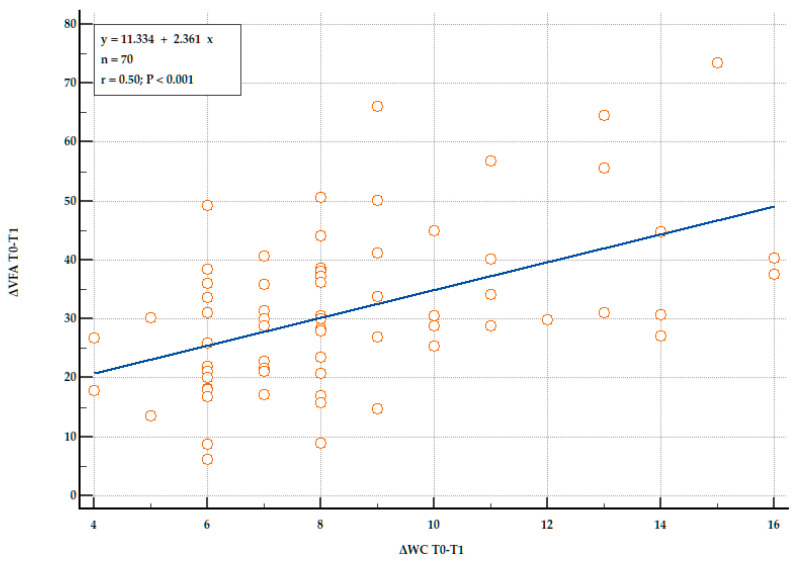
Scatter plot showing the relationship between changes in waist circumference (ΔWC) and visceral fat area (ΔVFA). The blue solid line represents the linear regression fit (r = 0.50, *p* < 0.001; R^2^ = 0.248; n = 70) and the red circles represent an individual participant..

A linear regression analysis was conducted to assess the association between changes in waist circumference (ΔWC) and body fat mass (ΔBFM). The results were statistically significant (β = 0.348, 95% CI [0.141–0.555], r = 0.38, *p* = 0.001), though the explained variance was modest (R^2^ = 0.142). This indicates that approximately 14.2% of the variance in body fat mass reduction is explained by changes in waist circumference, leaving substantial unexplained variability. In contrast, ΔWC showed a better correlation with ΔVFA (β = 2.360, 95% CI [1.367 to 3.354], r = 0.50, *p* < 0.001), suggesting that it is a more reliable proxy for changes in visceral fat than for total adiposity. Regression analysis indicated a steeper slope for ΔVFA than for ΔBFM, suggesting greater WC sensitivity to changes in visceral fat (Figure 4 and Figure 5). 

### 3.4. Effect of Semaglutide Between Groups

**Figure 6:**
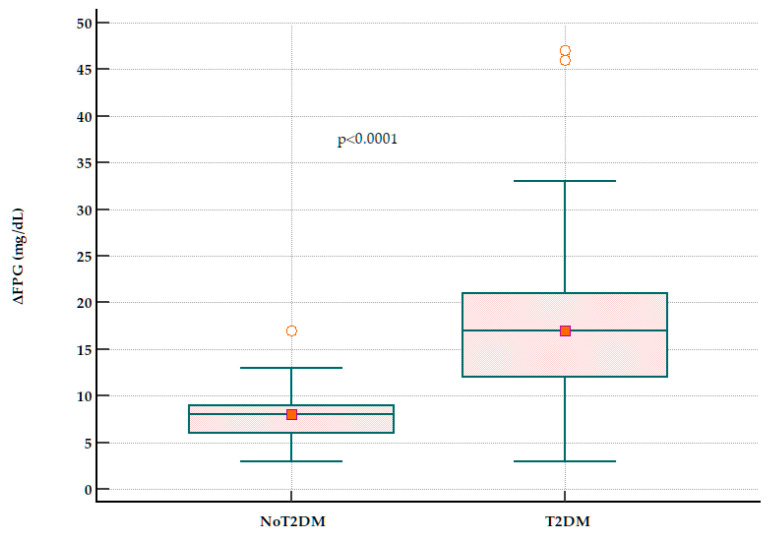
Comparison of FPG reductions between groups. The plot shows the median change (reduction) in FPG for each group. The diabetic group showed a significantly greater reduction than the non-diabetic group (*p* < 0.0001, Mann–Whitney U test). The median difference (Hodges–Lehmann shift) was −9.0 mg/dL.

**Figure 7:**
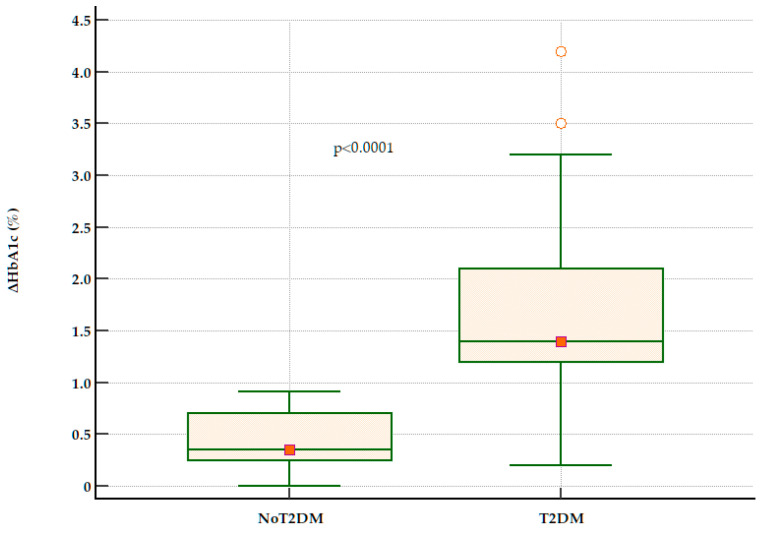
Comparison of HbA1c reduction between groups. The plot shows the median change (reduction) in HbA1c for each group. The diabetic group showed a significantly greater reduction than the non-diabetic group (*p* < 0.0001, Mann–Whitney U test), indicating substantial glycemic benefits in both groups. The median difference (Hodges–Lehmann shift) was −1.1%.

When stratified by diabetes status, both groups showed a significant decrease in FPG levels, with the non-diabetic group decreasing from a median of 103.5 mg/dL to 95.0 mg/dL and the diabetic group from a median of 127.5 mg/dL to 111.0 mg/dL (*p* < 0.0001, Wilcoxon signed-rank test). The treatment effect was significantly greater in the diabetic group than in the non-diabetic group. The median ΔFPG reduction was 17.0 mg/dL in the diabetic group, compared with 8.0 mg/dL in the non-diabetic group. The Hodges–Lehmann median shift was −9.0 mg/dL (95% CI: −11.0 to −6.0, *p* < 0.0001). A similar trend was observed for HbA1c: the non-diabetic group decreased from a median of 5.55% to 5.15%, and the diabetic group decreased from a median of 8.85% to 7.10% (*p* < 0.0001, Wilcoxon signed-rank test). The median ΔHbA1c (%) reduction was 1.4% in the diabetic group versus 0.35% in the non-diabetic group. The Hodges–Lehmann median shift was −1.1 (95% CI: −1.3 to −0.9, *p* < 0.0001) (Table 6). The distributions of ΔFPG and ΔHbA1c across both groups are shown in Figure 6 and Figure 7.

**Figure 8:**
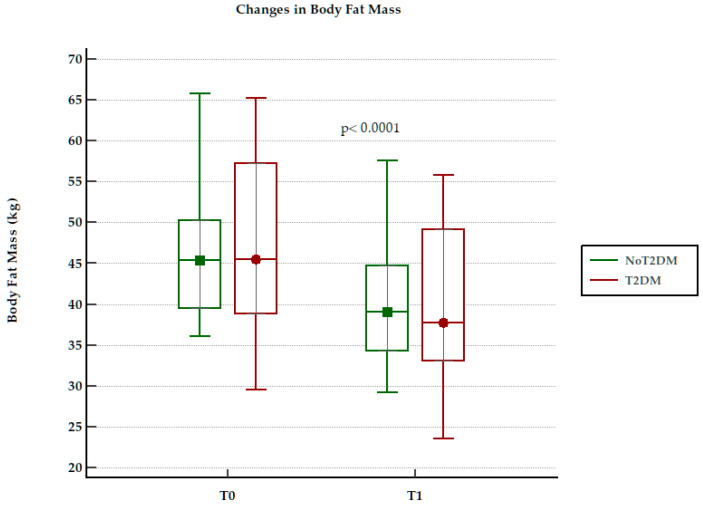
Longitudinal changes in body weight between and within groups across the two assessment points (T0, baseline; T1, follow-up). Body weight is presented as the median (square markers in the NoT2DM group and circles in the T2DM group) with 95% confidence intervals for the median (vertical bars).

**Figure 9:**
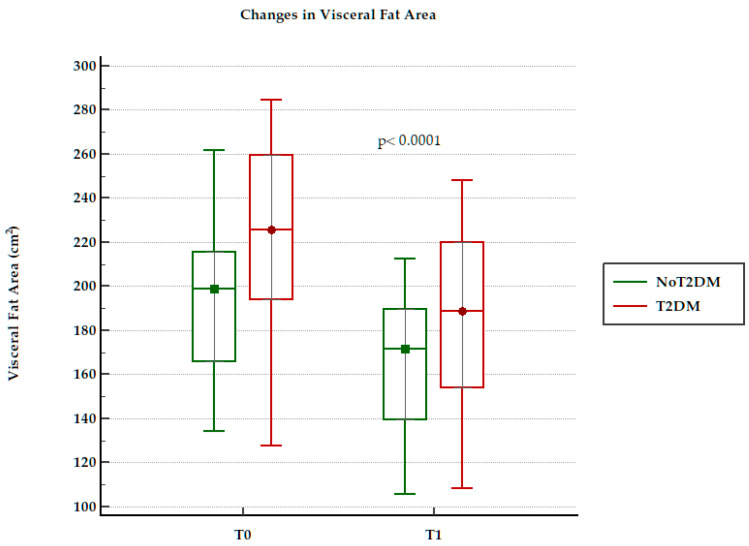
Longitudinal changes in visceral fat area (VFA) between groups across the two assessment points (T0, baseline; T1, follow-up). VFA is presented as the median (square markers in the NoT2DM group and circles in the T2DM group) with 95% confidence intervals for the median (vertical bars).

Additionally, significant reductions in body fat mass and visceral adipose tissue area were observed. The median decreases were −5.87 kg (95% CI: −6.45 to −5.3) in the non-diabetic group and −7.35 kg (95% CI: −8.3 to −6.45) in the diabetic group. The corresponding decreases in visceral adipose tissue area were −27.62 cm^2^ (95% CI: −32.65 to −23.05) in the non-diabetic group and −32.40 cm^2^ (95% CI: −36.30 to −28.80) in the diabetic group. SMM was largely preserved (−1.3 kg), indicating a favorable body composition response and a robust, homogeneous treatment effect on visceral adiposity (Figure 8 and Figure 9).

### 3.5. Correlation Analysis

Table 7 presents Spearman’s rank correlation analysis, revealing several significant associations among anthropometric and metabolic parameters. Strong positive correlations were observed between TyG index and TG/HDL (ρ = 0.659, *p* < 0.0001) and between METS-IR and TG/HDL (ρ = 0.764, *p* < 0.0001), indicating close relationships among lipid-related metabolic markers. Additionally, body weight showed strong correlations with BMI (ρ = 0.568, *p* < 0.0001) and WC (ρ = 0.507, *p* < 0.0001), reflecting the expected interdependence of anthropometric measures. Another strong correlation was observed between HbA1c and the TG/HDL ratio, TyG index, and WC (*p* < 0.0001), suggesting that poorer glycemic control is closely associated with insulin resistance, dyslipidemia, and central adiposity. Moderate correlations were identified between fat mass and BMI (ρ = 0.375, *p* = 0.0014) and between TyG index and the HOMA-IR (ρ = 0.352, *p* = 0.0028), suggesting partial associations between body composition and metabolic function. Inflammatory status, as measured by CRP, also showed a moderate correlation with HbA1c, indicating that worse glycemic control is associated with higher systemic inflammation, likely mediated by insulin resistance and metabolic dysfunction. In contrast, visceral fat area (VFA) showed weak, mostly non-significant correlations with other variables (e.g., weight: ρ = 0.015, *p* = 0.904), indicating a limited direct relationship within this dataset.

We performed a multivariate analysis for differences in weight, WC, VFA, FPG, HbA1c, using a stepwise variable selection (entry criterion *p* < 0.05, removal criterion *p* > 0.10) to identify independent predictors of treatment response. Only variables retained in the final models are presented in Table 8 for each outcome. All models included the entire cohort including participants with and without T2DM. The change in variables (∆) were calculated as follow-up value (T1) minus baseline value (T0). Positive values indicate increases, whereas negative values indicate decreases from baseline. All models showed acceptable variance inflation factors (VIF <3.0), indicating no significant multicollinearity among predictors. Age, diabetes duration, baseline BMI, and baseline body weight were tested but did not meet inclusion criteria in the final stepwise models.

## 4. Discussion

### 4.1. Interpretation of Findings

In this cohort of patients with obesity, semaglutide treatment led to consistent, clinically significant improvements across multiple cardiometabolic parameters. Weight loss was substantial, with nearly all participants achieving at least a 5% reduction from baseline, and was accompanied by marked reductions in waist circumference and visceral adiposity. Results on doses (maximum tolerated doses) and treatment duration suggest that the administered dose plays a more prominent role than treatment duration in influencing weight change, although the model’s moderate explanatory power indicates that other factors may also contribute. The more pronounced effect in the T2DM group may be explained by differences in treatment intensity, adherence, or underlying metabolic responsiveness. Additionally, although baseline characteristics were comparable, they may still have contributed to inter-individual variability in response.

Both study groups showed significant improvements in glycemic control over time, reflected by reductions in fasting glycemia and HbA1c from baseline (T0) to follow-up (T1). However, the magnitude of improvement differed between groups, with greater reductions observed in those with established diabetes. Baseline HbA1c emerged as the strongest predictor of HbA1c reduction, underscoring the importance of the initial glycemic burden in determining treatment response. Improvements in insulin resistance were closely linked to glycemic changes, supporting a mechanistic role for enhanced insulin sensitivity. These findings suggest that the intervention applied to patients with T2DM may provide superior efficacy in improving both short-term and long-term glycemic control. The greater reduction in HbA1c is particularly relevant clinically, as HbA1c reflects average glycemia over the previous 2–3 months and is strongly associated with the risk of microvascular and macrovascular complications.

Importantly, VFA decreased significantly, while SMM was largely preserved, indicating a favorable change in body composition. Given the strong association between visceral adiposity and metabolic risk, this finding is particularly relevant and may underlie several of the observed metabolic improvements. Changes in body mass index were comparable between groups, whereas decreases in body weight and waist circumference were slightly greater in the T2DM group.

Baseline values of each outcome variable emerged as the strongest associated factors of treatment-induced change, reflecting both regression to the mean and greater therapeutic potential in individuals with more severe metabolic impairment. Baseline VFA emerged as an independent predictor in both glycemic models (HbA1c and FPG), underscoring the central role of visceral adiposity as a modifiable driver of metabolic dysfunction. The substantially higher predictive power for glycemic outcomes (R^2^ = 0.61–0.79) compared to anthropometric outcomes (R^2^ = 0.22–0.25) suggests that glycemic response is more predictable based on baseline characteristics, whereas weight-related outcomes are influenced by unmeasured factors such as dietary adherence, physical activity, genetic variability, and concurrent medications.

Among the patients included in this study, a key observation was a significant reduction in CRP, indicating improved systemic inflammatory status. The large effect size suggests that semaglutide exerts meaningful anti-inflammatory effects, potentially mediated by reductions in HbA1c and metabolic stress, likely through insulin resistance and metabolic dysfunction.

Furthermore, surrogate indices of hepatic steatosis and fibrosis improved significantly after treatment, suggesting potential benefits for metabolic liver disease.

Together, these findings show that semaglutide may provide broad metabolic benefits in patients with obesity, regardless of diabetes status.

### 4.2. Strengths and Limitations

This study has several limitations that should be acknowledged. First, the relatively small sample size and the retrospective observational design may limit the generalizability of the findings and do not allow causal inference. Second, the lack of a control group, treatment variability (dose differences) and potential confounding from lifestyle interventions limit causal inference. The possibility of type I error related to multiple testing is acknowledged as a study limitation. In addition, potential confounding variables, including dietary habits, physical activity, treatment adherence, concomitant medications, and obesity-related comorbidities, were not specifically controlled or systematically evaluated, as these factors were beyond the primary scope of the study. The follow-up duration may also be insufficient to fully assess long-term metabolic outcomes and sustained glycemic control. Finally, variability in individual metabolic responses may have contributed to the dispersion of results despite the use of median-based statistical analyses.

Further large-scale, prospective, randomized controlled studies with longer follow-up periods are needed to confirm these findings and better clarify the determinants of metabolic response to semaglutide treatment.

### 4.3. Relation to Other Studies

Semaglutide has been shown to be effective not only in reducing body weight but also in improving overall metabolic and cardiovascular health. Clinical studies demonstrate that it induces significant and sustained weight loss (STEP 1—Semaglutide Treatment Effect in People with obesity, and STEP 5), provides meaningful metabolic benefits, improves glycemic control in patients with T2DM (STEP 2) [30], and reduces the risk of major adverse cardiovascular events (myocardial infarction and stroke) in individuals with obesity and established cardiovascular disease (SELECT). In addition, among patients with obesity-related heart failure, treatment has been associated with improved functional status and increased exercise capacity (STEP-HFpEF) [31].

Furthermore, semaglutide exerts important hepatoprotective effects, including reduced hepatic fat accumulation, resolution of hepatic inflammation in earlier stages of disease, and improvement in fibrosis in more advanced stages of MASLD. T2DM is increasingly recognized as a multifactorial condition associated with a significant neuropsychiatric burden, including heightened prevalence of anxiety, depression, and cognitive impairment that can progress to dementia. Concurrently, therapeutic agents such as semaglutide may modulate both metabolic and neuropsychiatric outcomes, while the susceptibility of this population to severe infectious diseases further underscores the complex interrelationship among metabolic dysregulation, mental health, and infection risk [11,32,33,34,35].

In the STEP 1 trial, Wilding and colleagues evaluated once-weekly semaglutide in adults with overweight or obesity without DM. The primary endpoint was weight loss, and the study showed a significant reduction in body weight compared with placebo. In addition to weight loss, semaglutide treatment led to improvements in key cardiometabolic measures, including reduced waist circumference, reflecting reduced visceral fat, and improved cardiovascular risk factors. These findings suggest that semaglutide improves overall metabolic health, not only body weight [2].

In clinical practice, long-term maintenance of weight loss is essential. The STEP 5 trial evaluated this by following patients for 104 weeks. Garvey and colleagues demonstrated that weight loss achieved with semaglutide 2.4 mg was sustained over two years, with continued metabolic benefits throughout the treatment period [36].

From a pathophysiological perspective, the primary mechanism is reduced visceral adipose tissue, a major determinant of metabolic dysfunction in T2DM. Reducing this fat compartment lowers free fatty acid flux to the liver, attenuates hepatic and skeletal muscle lipotoxicity, and, consequently, improves insulin signaling in peripheral tissues. As a result, a metabolic “cascade” may occur, characterized by simultaneous improvements in glycemic control, reductions in triglyceride levels, and decreased systemic inflammation, parameters that are closely associated with cardiovascular risk and the progression of obesity-related metabolic disease [37,38].

In the STEP 2 study, conducted in patients with obesity or overweight and T2DM, treatment with semaglutide 2.4 mg once weekly was associated with significantly greater weight loss than placebo and improved glycemic control, supporting its role as a therapeutic option for the integrated management of obesity and T2DM [39].

### 4.4. Relevance of Our Findings

Our results highlight the cardiometabolic benefits of semaglutide in obesity. While the overall cohort showed broad improvements from baseline to follow-up, effects in individuals without T2DM were primarily limited to reductions in FPG, HbA1c, body weight, and VFA. This may suggest a differential glycemic status-dependent impact, supporting a role for semaglutide in targeted reduction of cardiometabolic risk.

## 5. Conclusions

In conclusion, semaglutide treatment was associated with significant improvements in body weight, glycemic control, body composition, insulin resistance, and cardiometabolic risk markers in people with obesity, with and without T2DM.

Baseline metabolic characteristics, particularly visceral adiposity and glycemic status, may influence treatment response and support a more individualized approach to obesity management.

## Figures and Tables

**Table 1:** Definitions of Hepatic, Metabolic, and Insulin Resistance Indices.

Biomarker/Index	Category	Definition	Cut-Off Value	Reference
HSI (Hepatic Steatosis Index)	Hepatic steatosis index	Calculated based on ALT/AST ratio, BMI, sex, and presence of diabetes	<30 (exclude NAFLD), >36 (suggest NAFLD)	[16]
FLI (Fatty Liver Index)	Hepatic steatosis index	Algorithm based on BMI, waist circumference, triglycerides, and GGT	<30 (rule-out NAFLD/MASLD), ≥60 (rule-in NAFLD/MASLD)	[17,18]
FIB-4 (fibrosis-4)	Liver fibrosis index	Calculated from age, AST, ALT, and platelet count	<1.3 or 1.45 (low risk), >2.67 or 3.25 (high risk fibrosis)	[19]
HOMA-IR (Homeostatic Model Assessment for Insulin Resistance)	Insulin resistance index	Calculated from fasting glucose and insulin levels	>2.0–2.5 (insulin resistance, population-dependent)	[20,21,22,23]
TyG index (triglycerides-glucose index)	Insulin resistance surrogate index	Derived from fasting triglycerides and glucose	No universal cut-off; ~≥8.8–9.0 (population-dependent)	[24,25,26]
METS-IR (Metabolic Score for Insulin Resistance)	Insulin resistance index	Composite metabolic score including glucose and lipid parameters	No universal cut-off; ~30–50 used in the literature (population-dependent)	[27]
TG/HDL ratio	Atherogenic/metabolic risk marker	Ratio of triglycerides to HDL cholesterol	>3.0–3.5 indicates increased insulin resistance risk	[28,29]

The cut-off values were derived from previously validated studies. ALT—alanine aminotransferase; AST—aspartate aminotransferase; NAFLD—non-alcoholic fatty liver disease; BMI—body mass index; GGT—gamma-glutamyl transferase; MASLD—metabolic dysfunction-associated steatotic liver disease; TG—triglycerides; HDL—high-density lipoprotein.

**Table 2:** Baseline characteristics of the study population, stratified by T2DM status.

Variable		NoT2DM (*n* = 28)	T2DM (*n* = 42)	*p*-Value
Age (years) ^†^		42 (36.5–46.5)	60 (54–62)	<0.0001 ***
Sex ^††^	Male, *n* (%)	8 (28.6%)	14 (33.3%)	0.67 ^ns^
	Female, *n* (%)	20 (71.4%)	28 (66.7%)	
Weight (kg) ^†^		96.5 (90.0–108.5)	110 (98–116)	0.014 *
BMI (kg/m^2^) ^†^		33 (1–35)	38 (37–41)	<0.0001 ***
WC (cm) ^†^		100.5 (94.0–107.0)	113.5 (102.0–119.0)	<0.0001 ***
SBP (mmHg) ^†^		127 (122.0–131.5)	142 (132–150)	<0.0001 ***
DBP (mmHg) ^†^		88 (86.5–90.0)	95 (92–99)	<0.0001 ***
FPG (mg/dL) ^†^		103.5 (95.5–127.5)	127.5 (121.0–136.0)	<0.0001 ***
HbA1c (%) ^†^		5.55 (5.35–5.85)	8.85 (7.90–9.60)	<0.0001 ***
LDLc (mg/dL) ^†^		146.5 (119.5 to 155.0)	153.5 (131.0–172.0)	0.11 ^ns^
TG (mg/dL) ^†^		185 (150–199)	240.5 (200.0–270.0)	<0.0001 ***
HDLc (mg/dL) ^†^		44.5 (39.5–52.0)	37 (33–41)	<0.0001 ***
ALT (U/L) ^†^		35.0 (28.5–43.0)	30 (15–35)	0.09 ^ns^
AST (U/L) ^†^		31 (24–36)	29 (21–35)	0.54 ^ns^
GGT (U/L) ^†^		43.5 (38.5–50.5)	40 (34–55)	0.34 ^ns^
ALB (g/dL) ^†^		5.45 (4.55–6.10)	5.50 (5.10–6.20)	0.41 ^ns^
CRP (mg/L) ^†^		2.85 (2.35–3.25)	3.45 (2.50–4.40)	0.02 *
sCr (mg/dL) ^†^		0.75 (0.70–0.80)	0.88 (0.78–0.95)	0.0004 ***
eGFR (ml/min) ^†^		104.0 (96.5–118.0)	83.5 (72.0–93.0)	<0.0001 ***
uACR (mg/g) ^†^		15.5 (12.0–18.5)	30.0 (20.0–37.0)	<0.0001 ***
Uric acid (mg/dL) ^†^		5.75 (5.20–6.05)	6.05 (5.10–6.80)	0.18 ^ns^
PLT (x10^9^/ L)		275 (241–300)	278 (237–301)	1.00 ^ns^
BFM (Kg) ^†^		45.35 (39.50–50.25)	45.55 (38.90–57.30)	0.65 ^ns^
SMM (Kg) ^†^		28.80 (26.65–34.00)	30.10 (28.40–33.20)	0.32 ^ns^
VFA (cm^2^) ^†^		198.8 (166.1–215.8)	225.8 (194.1–259.6)	0.01 *
HSI ^†^		43.20 (41.65–47.95)	50.00 (47.50–53.80)	<0.0001 ***
FLI ^†^		85.5 (77.0–92.0)	97.0 (92.0–98.0)	<0.0001 ***
FIB-4^†^		0.68 (0.58–0.85)	1.19 (0.91–1.51)	<0.0001 ***
HOMA-IR ^†^		5.60 (4.65–6.40)	5.20 (4.30–6.10)	0.49 ^ns^
TyG index ^†^		1.81 (1.64–1.90)	1.83 (1.45–2.03)	0.24 ^ns^
METS-IR ^†^		50.75 (48.25–54.65)	67.10 (62.30–70.90)	<0.0001 ***
TG/HDL ratio ^†^		4.31 (2.74–4.96)	6.18 (5.39–7.30)	<0.0001 ***

^†^ Mann–Whitney test; ^††^ chi-squared test. In this study, a two-sided *p*-value < 0.05 was considered the threshold for statistical significance (^ns^ = not significant, *p* ≥ 0.05; * *p* < 0.05; *** *p* < 0.001). Values are expressed as the median (IQR) for non-normally distributed variables. Abbreviations: IQR = interquartile range; T2DM = type 2 diabetes mellitus; BMI = body mass index; WC = waist circumference; SBP = systolic blood pressure; DBP = diastolic blood pressure; FPG = fasting plasma glucose; LDLc = low-density lipoprotein; TG = triglyceride; HDL c = high-density lipoprotein; ALT = alanine aminotransferase; AST = aspartate aminotransferase; GGT = gamma-glutamyl Transferase; ALB = serum albumin; CRP = C-reactive protein; sCr = serum creatinine; eGFR = estimated glomerular filtration rate; uACR = urinary albumin–creatinine ratio; PLT = platelet count; BFM = Body Fat Mass; SMM = Skeletal Muscle Mass; VFA = Visceral Fat Area; HSI = Hepatic Steatosis Index; FLI = Fatty Liver Index; FIB-4 = fibrosis-4; HOMA-IR = Homeostatic Model Assessment for Insulin Resistance; TyG = triglycerides-glucose; METS-IR = Metabolic Score for Insulin Resistance.

**Table 3:** Doses of treatment and number of patients in both groups.

Doses	NoT2DM (*n* = 28)	T2DM (*n* = 42)	n (%)	*p*-Value ^††^
0.5 mg (*n*)	1	2	3 (4.3)	0.21 ^ns^
1 mg (*n*)	8	5	13 (18.6)	
1.7 mg (*n*)	19	35	54 (77.1)	
			n = 70	

^††^ Chi-square test of independence. A two-sided *p*-value < 0.05 was considered the threshold for statistical significance (^ns^ = not significant, *p* ≥ 0.05.

**Table 4:** Changes in percentage weight loss after treatment.

Variable	Weight Loss					
	>5%		*p*-Value ^††^	>10%		*p*-Value ^††^
	No	Yes		No	Yes	
NoT2DM (%)	0.0	100.0	0.24 ^ns^	67.9	32.1	0.14 ^ns^
T2DM (%)	4.8	95.2		50.0	50.0	
Male (%)	4.5	95.5	0.56 ^ns^	63.6	36.4	0.46 ^ns^
Female (%)	2.1	97.9		54.2	45.8	

^††^ Chi-squad test of independence. A two-sided *p*-value < 0.05 was considered the threshold for statistical significance (^ns^ = not significant, *p* ≥ 0.05. Responders were defined as those who achieved >5% or >10% weight loss from baseline.

**Table 5:** Changes from baseline (T0) to follow-up (T1) in anthropometric, metabolic, cardiovascular, hepatic, and body composition parameters.

Variable		Median Value		*p*-Value ^‡^
	T0 (*n* = 70)	T1 (*n* = 70)	Δ	
Weight (kg)	106.5	96.5	−9.0	<0.0001 ***
BMI (kg/m^2^)	37	33	−4.0	<0.0001 ***
WC (cm)	108.0	97.0	−8.0	<0.0001 ***
SBP (mmHg)	148.5	135.0	−7.5	<0.0001 ***
DBP (mmHg)	92.0	85.0	−12.0	<0.0001 ***
FPG (mg/dL)	119.0	105.0	−12.0	<0.0001 ***
HbA1c (%)	7.8	6.6	−1.05	<0.0001 ***
LDLc (mg/dL)	150.0	120.0	−28.0	<0.0001 ***
TG (mg/dL)	203.5	158.5	−43.0	<0.0001 ***
HDLc (mg/dL)	39.0	49.0	10.0	<0.0001 ***
ALT (U/L)	31.0	25.0	−5.0	<0.0001 ***
AST (U/L)	29.0	22.5	−6.0	<0.0001 ***
GGT (U/L)	42.0	31.0	−10.0	<0.0001 ***
ALB (g/dL)	5.5	5.3	−0.1	0.16 ^ns^
CRP (mg/L)	3.1	2.1	−0.8	<0.0001 ***
sCr (mg/dL)	0.8	0.7	−0.07	<0.0001 ***
eGFR (ml/min)	93.0	100.0	7.0	<0.0001 ***
uACR (mg/g)	21.0	17.0	−5.0	<0.0001 ***
Uric acid (mg/dL)	5.8	5.3	−0.4	<0.0001 ***
BFM (Kg)	45.4	38.1	−6.6	<0.0001 ***
SMM (Kg)	29.8	28.7	−1.3	<0.0001 ***
VFA (cm^2^)	205.6	180.4	−30.1	<0.0001 ***
HSI	48.1	44.7	−3.6	<0.0001 ***
FLI	93.0	79.0	−14.5	<0.0001 ***
FIB-4	0.90	0.84	−0.06	<0.0001 ***
HOMA-IR	5.3	2.9	−2.3	<0.0001 ***
TyG index	1.80	1.56	−0.19	<0.0001 ***
METS-IR	60.6	51.2	−10.3	<0.0001 ***
TG/HDL ratio	5.38	3.22	−1.93	<0.0001 ***

^‡^ Wilcoxon test. In this study, a two-sided *p*-value < 0.05 was considered the threshold for statistical significance (^ns^ = not significant, *p* ≥ 0.05; *** *p* < 0.001). Values are expressed as the median (IQR) for non-normally distributed variables. Abbreviations: HbA1c = hemoblogin A1c; Δ = Difference between T0 and T1; BMI = body mass index; WC = waist circumference; SBP = systolic blood pressure; DBP = diastolic blood pressure; FPG = fasting plasma glucose; LDLc = low-density lipoprotein; TG = triglyceride; HDLc = high-density lipoprotein; ALT = alanine aminotransferase; AST = aspartate aminotransferase; GGT = gamma-glutamyl Transferase; ALB = serum albumin; CRP = C-reactive protein; sCr = serum creatinine; eGFR = estimated glomerular filtration rate; ACR = albumin–creatinine ratio; BFM = Body Fat Mass; SMM = Skeletal Muscle Mass; VFA = Visceral Fat Area; HSI = Hepatic Steatosis Index; FLI = Fatty Liver Index; FIB-4 = fibrosis-4; HOMA-IR = Homeostatic Model Assessment for Insulin Resistance; TyG = triglycerides-glucose; METS-IR = Metabolic Score for Insulin Resistance;.

**Table 6:** Comparison of median changes in FPG and HbA1c between and within study groups from T0 to T1 and of the Δ reduction.

Variable	No T2DM (*n* = 28)	T2DM (*n* = 42)	*p*-Value (Between Groups) ^†^
FPG T0 (mg/dL)	103.5 (95.5–107.0)	127.5 (121.0–136.0)	<0.0001 ***
FPG T1 (mg/dL)	95.0 (86.0–98.0)	111.0 (107.0–117.0)	<0.0001 ***
	*p*-Value (within group) ^‡^		
	*p* < 0.0001 ***	*p* < 0.0001 ***	
ΔFPG (mg/dL)	8.0 (6.0–9.0)	17.0 (12.0–21.0)	<0.0001 ***
			
**Variable**	**No T2DM** **(*n* = 28)**	**T2DM** **(*n* = 42)**	***p*-Value (between groups) ^†^**
HbA1c T0 (%)	5.55 (5.35–5.85)	8.85 (7.90–9.60)	<0.0001 ***
HbA1c T1 (%)	5.15 (5.05–5.30)	7.10 (6.80–7.70)	<0.0001 ***
	*p*-Value (within group) ^‡^		
	*p* < 0.0001 ***	*p* < 0.0001 ***	
			
ΔHbA1c (%)	0.35 (0.25–0.70)	1.4 (1.2–2.1)	<0.0001 ***

^†^ Mann–Whitney U test; Hodges–Lehmann median shift (95% CI). ^‡^ Wilcoxon signed-rank test. Data are presented as median and interquartile ranges [IQR]. A two-sided *p*-value < 0.05 was considered the threshold for statistical significance (*** *p* < 0.001). Data normality was assessed using the Shapiro–Wilk test.

**Table 7:** Spearman correlation matrix of changes (Δ = follow-up − baseline) in anthropometric and metabolic parameters. Warmer colors indicate stronger positive correlations. Each cell reports Spearman’s ρ, *p*-value, and sample size (n = 70).

ΔMETS-IR	1	0.764 *p* < 0.0001 n = 70	0.601 *p* < 0.0001 n = 70	0.556 *p* < 0.0001 n = 70	0.403 *p* = 0.0005 n = 70	0.455 *p* = 0.0001 n = 70	0.385 *p* = 0.0010 n = 70	0.691 *p* < 0.0001 n = 70	0.277 *p* = 0.0202 n = 70	0.172 *p* = 0.1535 n = 70	0.100 *p* = 0.4092 n = 70	−0.034 *p* = 0.7815 n = 70
ΔTG/HDL ratio	0.764 *p* < 0.0001 n = 70	1	0.659 *p* < 0.0001 n = 70	0.434 *p* = 0.0002 n = 70	0.388 *p* = 0.0009 n = 70	0.491 *p* < 0.0001 n = 70	0.303 *p* = 0.0107 n = 70	0.196 *p* = 0.1043 n = 70	0.368 *p* = 0.0017 n = 70	0.081 *p* = 0.5058 n = 70	0.174 *p* = 0.1492 n = 70	0.004 *p* = 0.9731 n = 70
ΔTyG index	0.601 *p* < 0.0001 n = 70	0.659 *p* < 0.0001 n = 70	1	0.404 *p* = 0.0005 n = 70	0.371 *p* = 0.0016 n = 70	0.480 *p* < 0.0001 n = 70	0.308 *p* = 0.0096 n = 70	0.308 *p* = 0.0094 n = 70	0.352 *p* = 0.0028 n = 70	0.055 *p* = 0.6515 n = 70	0.241 *p* = 0.0446 n = 70	−0.171 *p* = 0.1576 n = 70
ΔWeight	0.556 *p* < 0.0001 n = 70	0.434 *p* = 0.0002 n = 70	0.404 *p* = 0.0005 n = 70	1	0.507 *p* < 0.0001 n = 70	0.341 *p* = 0.0038 n = 70	0.485 *p* < 0.0001 n = 70	0.568 *p* < 0.0001 n = 70	0.236 *p* = 0.0495 n = 70	0.015 *p* = 0.9040 n = 70	0.174 *p* = 0.1501 n = 70	−0.103 *p* = 0.3950 n = 70
ΔWC	0.403 *p* = 0.0005 n = 70	0.388 *p* = 0.0009 n = 70	0.371 *p* = 0.0016 n = 70	0.507 *p* < 0.0001 n = 70	1	0.478 *p* < 0.0001 n = 70	0.440 *p* = 0.0001 n = 70	0.291 *p* = 0.0145 n = 70	0.312 *p* = 0.0086 n = 70	0.484 *p* < 0.0001 n = 70	0.263 *p* = 0.0278 n = 70	0.118 *p* = 0.3320 n = 70
ΔHbA1c	0.455 *p* = 0.0001 n = 70	0.491 *p* < 0.0001 n = 70	0.480 *p* < 0.0001 n = 70	0.341 *p* = 0.0038 n = 70	0.478 *p* < 0.0001 n = 70	1	0.408 *p* = 0.0005 n = 70	0.174 *p* = 0.1492 n = 70	0.226 *p* = 0.0598 n = 70	0.295 *p* = 0.0132 n = 70	0.357 *p* = 0.0024 n = 70	0.027 *p* = 0.8233 n = 70
ΔBFM	0.385 *p* = 0.0010 n = 70	0.303 *p* = 0.0107 n = 70	0.308 *p* = 0.0096 n = 70	0.485 *p* < 0.0001 n = 70	0.440 *p* = 0.0001 n = 70	0.408 *p* = 0.0005 n = 70	1	0.375 *p* = 0.0014 n = 70	0.128 *p* = 0.2904 n = 70	0.397 *p* = 0.0007 n = 70	0.242 *p* = 0.0438 n = 70	−0.086 *p* = 0.4769 n = 70
ΔBMI	0.691 *p* < 0.0001 n = 70	0.196 *p* = 0.1043 n = 70	0.308 *p* = 0.0094 n = 70	0.568 *p* < 0.0001 n = 70	0.291 *p* = 0.0145 n = 70	0.174 *p* = 0.1492 n = 70	0.375 *p* = 0.0014 n = 70	1	0.056 *p* = 0.6452 n = 70	0.170 *p* = 0.1589 n = 70	−0.010 *p* = 0.9352 n = 70	−0.183 *p* = 0.1288 n = 70
ΔHOMA	0.277 *p* = 0.0202 n = 70	0.368 *p* = 0.0017 n = 70	0.352 *p* = 0.0028 n = 70	0.236 *p* = 0.0495 n = 70	0.312 *p* = 0.0086 n = 70	0.226 *p* = 0.0598 n = 70	0.128 *p* = 0.2904 n = 70	0.056 *p* = 0.6452 n = 70	1	0.195 *p* = 0.1064 n = 70	0.015 *p* = 0.9025 n = 70	0.113 *p* = 0.3517 n = 70
ΔVFA	0.172 *p* = 0.1535 n = 70	0.081 *p* = 0.5058 n = 70	0.055 *p* = 0.6515 n = 70	0.015 *p* = 0.9040 n = 70	0.484 *p* < 0.0001 n = 70	0.295 *p* = 0.0132 n = 70	0.397 *p* = 0.0007 n = 70	0.170 *p* = 0.1589 n = 70	0.195 *p* = 0.1064 n = 70	1	−0.043 *p* = 0.7268 n = 70	0.108 *p* = 0.3733 n = 70
ΔCRP	0.100 *p* = 0.4092 n = 70	0.174 *p* = 0.1492 n = 70	0.241 *p* = 0.0446 n = 70	0.174 *p* = 0.1501 n = 70	0.263 *p* = 0.0278 n = 70	0.357 *p* = 0.0024 n = 70	0.242 *p* = 0.0438 n = 70	−0.010 *p* = 0.9352 n = 70	0.015 *p* = 0.9025 n = 70	−0.043 *p* = 0.7268 n = 70	1	0.033 *p* = 0.7851 n = 70
ΔFIB−4	−0.034 *p* = 0.7815 n = 70	0.004 *p* = 0.9731 n = 70	−0.171 *p* = 0.1576 n = 70	−0.103 *p* = 0.3950 n = 70	0.118 *p* = 0.3320 n = 70	0.027 *p* = 0.8233 n = 70	−0.086 *p* = 0.4769 n = 70	−0.183 *p* = 0.1288 n = 70	0.113 *p* = 0.3517 n = 70	0.108 *p* = 0.3733 n = 70	0.033 *p* = 0.7851 n = 70	1
	ΔMETS-IR	ΔTG/HDL ratio	ΔTyG index	ΔWeight	ΔWC	ΔHbA1c	ΔBFM	ΔBMI	ΔHOMA	ΔVFA	ΔCRP	ΔFIB−4

**Table 8:** Multivariate regression analysis of associated factors with treatment response to semaglutide.

Outcome Variable	Predictor	β Coefficient	SE	95% CI	t-Statistic	*p*-Value	Model Statistics
∆ Weight (kg)							Adjusted R^2^ = 0.228
	Baseline WC	0.128	0.028	0.073 to 0.184	4.62	<0.0001	F = 21.32
	Constant	−4.204	2.983	−10.156 to 1.748	−1.41	0.163	*p* < 0.0001
∆ WC (cm)							Adjusted R^2^ = 0.257
	Baseline WC	0.135	0.027	0.081 to 0.189	4.98	<0.0001	F = 24.82
	Constant	−5.967	2.916	−11.784 to −0.149	−2.05	0.045	*p* < 0.0001
∆ HbA1c (%)							Adjusted R^2^ = 0.799
	Baseline HbA1c	0.452	0.037	0.377 to 0.526	12.15	<0.0001	F = 92.34
	Baseline VFA	0.004	0.001	0.002 to 0.007	3.39	0.001	*p* < 0.0001
	Baseline FPG	−0.009	0.004	−0.017 to −0.001	−2.37	0.021	
	Constant	−2.066	0.392	−2.850 to −1.283	−5.27	<0.0001	
∆ FPG (mg/dL)							Adjusted R^2^ = 0.613
	Baseline FPG	0.453	0.051	0.352 to 0.554	8.97	<0.0001	F = 37.41
	Baseline VFA	0.047	0.017	0.014 to 0.081	2.86	0.006	*p* < 0.0001
	Baseline HbA1c	−1.484	0.491	−2.465 to −0.502	−3.02	0.004	
	Constant	−38.288	5.185	−48.640 to −27.937	−7.38	<0.0001	
∆ VFA (cm^2^)							Adjusted R^2^ = 0.150
	Baseline VFA	0.114	0.031	0.051 to 0.177	3.63	0.001	F = 13.18
	Constant	4.949	6.732	−8.484 to 18.382	0.74	0.465	*p* = 0.0005

Abbreviations: ∆ = Change (delta) from baseline to follow-up; β = Standardized regression coefficient; CI = Confidence interval; F = F-statistic from ANOVA for overall model significance; FPG = Fasting plasma glucose; HbA1c = Glycated hemoglobin; *p* = Probability value (two-tailed); R^2^ = Coefficient of determination (proportion of variance explained); SE = Standard error; VFA = Visceral fat area (measured by bioelectrical impedance analysis); WC = Waist circumference.

## Data Availability

The data presented in this study are available upon request from the corresponding author.

## Abbreviations

The following abbreviations are used in this manuscript:

ALT	Alanine aminotransferase
AST	Aspartate aminotransferase
cAMP	Cyclic adenosine monophosphate
CART	Cocaine-and amphetamine-regulated transcript
ChREBP	Carbohydrate-responsive element-binding protein
CKD	Chronic kidney disease
CVD	Cardiovascular disease
DM	Diabetes mellitus
GGT	Gamma-glutamyl transferase
GLP-1RA	Glucagon-like peptide-1 receptor agonist
IR	Insulin resistance
LDL	Low-Density Lipoprotein
MASLD	Metabolic Dysfunction-Associated Steatotic Liver Disease
MS	Metabolic syndrome
NAFLD	Non-alcoholic fatty liver disease
NCDs	Noncommunicable diseases
NPY/AgRP	Neuropeptide Y/agouti-related peptide
PKA	Protein kinase A
POMC	Proopiomelanocortin
ROS	Reactive oxygen species
SELECT	Semaglutide Effects on Cardiovascular Outcomes in People with Overweight or Obesity
SREBP-1c	Sterol regulatory element-binding protein-1c
STEP	Semaglutide Treatment Effect in People with obesity
STROBE	Strengthening the Reporting of Observational Studies in Epidemiology
SUSTAIN	Semaglutide Unabated Sustainability in Treatment of Type 2 Diabetes
T2DM	Type 2 diabetes mellitus
